# *De Novo* Transcriptome Assembly and Analysis of the Flat Oyster Pathogenic Protozoa *Bonamia Ostreae*

**DOI:** 10.3389/fcimb.2022.921136

**Published:** 2022-07-14

**Authors:** Germain Chevignon, Aurélie Dotto-Maurel, Delphine Serpin, Bruno Chollet, Isabelle Arzul

**Affiliations:** Ifremer, ASIM, La Tremblade, France

**Keywords:** *Bonamia ostreae*, protozoan parasite, Haplosporida, RNAseq, *Ostrea edulis*, flat oyster, oyster

## Abstract

The flat oyster *Ostrea edulis* is an oyster species native to Europe. It has declined to functional extinction in many areas of the NE Atlantic for several decades. Factors explaining this decline include over-exploitation of natural populations and diseases like bonamiosis, regulated across both the EU and the wider world and caused by the intracellular protozoan parasite *Bonamia ostreae.* To date, very limited sequence data are available for this Haplosporidian species. We present here the first transcriptome of *B. ostreae*. As this protozoan is not yet culturable, it remains extremely challenging to obtain high-quality *-omic* data. Thanks to a specific parasite isolation protocol and a dedicated bioinformatic pipeline, we were able to obtain a high-quality transcriptome for an intracellular marine micro-eukaryote, which will be very helpful to better understand its biology and to consider the development of new relevant diagnostic tools.

## Introduction

The flat oyster, *Ostrea edulis*, is native to Europe. Present along the European coasts, it was translocated to the USA in the 1940s and 1950s. Its production has severely been affected by overfishing and by diseases such as bonamiosis due to the protozoan parasite *Bonamia ostreae* (Buestel et al., 2009). First described in 1979 in the context of a flat oyster mortality outbreak in L’Ile Tudy, Brittany, France (Pichot et al., 1979), it has spread to most European oyster-producing areas, Canada, and the USA through aquaculture-related movements (Pogoda et al., 2019). *B. ostreae* has also recently been detected in the Chilean oyster *Ostrea chilensis* in New Zealand (Lane et al., 2016). This 2–3-µm microparasite multiplies within hemocytes, bivalve circulating cells notably involved in defense mechanisms against pathogens. Infected oysters may present macroscopical signs such as tissue discoloration, gill indentation, or ulcers. However, most infected individuals are asymptomatic. The multiplication of the parasite within hemocytes leads to their destruction and finally host death (Engelsma et al., 2014).

Considering its impact on farmed and natural populations of flat oysters, *Bonamia ostreae* is regulated and notifiable to the European Union and the World Organization of Animal Health. In this context, geographic zones hosting *Ostrea edulis* are subject to surveillance and control programs.

*Bonamia ostreae* belongs to the Haplosporida Order and the Haplosporiidae family. This family includes species of the genera *Bonamia*, *Haplosporidium*, and *Minchinia*. Based on ultrastructural and molecular information, to date, four *Bonamia* species have been described (*B. ostreae*, *B. exitiosa*, *B. perspora*, and *B. roughleyi* (Arzul and Carnegie, 2015), although the identity of *B. roughleyi* remains uncertain (Carnegie et al., 2014). Only reported in Northern America and Australia, respectively, *B. perspora* and *B. roughleyi* have never been detected in Europe, whereas *B. ostreae* and *B. exitiosa* are present and might even be sympatric in some flat oyster populations in several European countries (Abollo et al., 2008; Arzul, 2018).

The diagnosis of bonamiosis has for a long time relied on histology and cytology (heart or gill imprints). However, the sensitivity of these tools is low, and they do not permit parasite identification at the species level or distinguishing between close parasites such as *B. ostreae* and *B. exitiosa*. Molecular tools, including conventional and real-time PCR, are also available. Some of these assays amplify not only *B. ostreae* but also other members of the family Haplosporiidae. Nevertheless, some recently developed real-time PCRs allow rapid and specific detection of *B. ostreae* (Robert et al., 2009; Ramilo et al., 2013).

**Figure 1:**
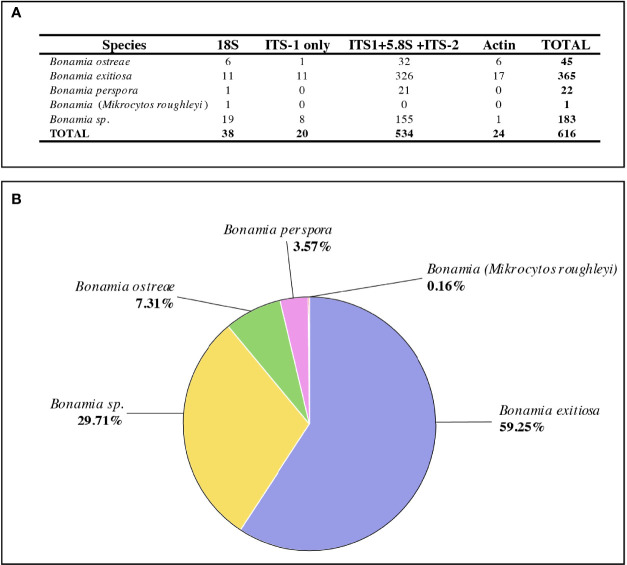
Overview of Bonamia sequence data available in public databases. **(A)** Number of nucleotide sequences available in NCBI for protists of the genus Bonamia. (Source: NCBI, August 03, 2021, Query: Bonamia, output has been checked, removing nonrelevant sequences). **(B)** Percentages of nucleotide sequences available for protists of the genus Bonamia in NCBI by species (Source: NCBI, August 03, 2021, Query: Bonamia, output has been checked, removing nonrelevant sequences).

Nucleotide data available in public databases for parasites of the genus Bonamia are scarce and mostly concern Bonamia exitiosa (59%) and Bonamia sp. (30%). B. ostreae represents only 7% of sequences currently available (**Figure 1**). These B. ostreae sequences are partial small subunit ribosomal RNA gene sequences (13%); internal transcribed spacer 1–5.8S–internal transcribed spacer 2 sequences (72%); and partial actin gene sequences (13%) (**Figure 1**). In addition, a few amino acid sequences have been characterized, including two actin genes (López-Flores et al., 2007) and a heat shock protein 90 involved in the internalization of the parasite within hemocytes (Prado-Alvarez et al., 2013).

The low sequence information available is notably explained by the fact that micro-eukaryote parasites such as *Bonamia* parasites are non-cultivable and difficult to obtain from infected hosts. In this context, our goal was to increase the number of sequences available for *Bonamia ostreae*. For this purpose and considering the lack of a reference genome as well as the possible presence of bacteria contaminating our parasite suspensions, we have decided to sequence RNA extracted from *B. ostreae* parasites isolated from highly infected flat oysters using Illumina. The obtained results allowed us to not only assemble and analyze rRNA reads but also identify and characterize the entire *B. ostreae* rRNA operon. In complement, to confirm the obtained sequence, primers were designed to amplify a large part of the rRNA operon, which was subsequently sequenced using ONT from DNA extracted from infected oysters.

To our knowledge, this is the first time RNA sequencing (RNAseq) has been used to improve our knowledge of the protozoan parasite *Bonamia ostreae*. Our results have drastically improved the number of sequences available for this regulated parasite species, allowing us to better understand its biology, in particular its interactions with its host, and to consider the development of new relevant diagnostic tools.

## Materials and Methods

### Selection of Flat Oysters Highly Infected With Bonamia ostreae

Adult flat oysters *Ostrea edulis* collected in 2012 from Quiberon Bay, Brittany (France), were maintained in Ifremer La Tremblade facilities until being screened for the presence of *Bonamia ostreae* by imprint cytology of heart tissue. Two oysters found highly infected were selected and immediately processed for parasite isolation.

### Parasite Isolation

Using an Ultra-Turrax homogenizer, all the organs except the adductor muscle were homogenized, and the parasites were concentrated by differential centrifugation on sucrose gradients and then purified by isopycnic centrifugation on a Percoll gradient (Mialhe et al., 1988). Isolated parasites were suspended in filtered seawater (FSW) and counted using a Malassez cell chamber. Finally, 2 × 10^8^ parasites were fixed in Trizol (Invitrogen, USA) and stored at −80°C until being processed for RNA extraction.

### RNA Extraction and Control

RNA extraction was carried out according to the Trizol manufacturer’s recommendations. RNA was subsequently digested with Dnase TURBO (Ambion)—purity (260/280 = 1.9 and 260/230 = 0.63) and concentration (232 ng/µl) of RNA was estimated using the NanoDrop spectrophotometer. The integrity of RNA was also checked using Agilent RNA6000 Nano Kit on an Agilent 2100 Bioanalyzer pRIN = 6.5; 28S/18S not determined; these parameters vary depending on species (Gayral et al., 2011)]. Although all recommended criteria were not met (RNA concentration >100 ng/µl; 260/280 and 260/230 > 1.8; RIN > 8.5 28S/18S > 1.8), the RNA suspension was used to prepare libraries as described below.

In addition, the RNA suspension was tested in duplicate by real-time PCR targeting *Bonamia ostreae* actin 1 gene (Robert et al., 2009) following the authors’ recommendations. No *B. ostreae* was detected, confirming the absence of parasite DNA in the RNA suspension. The absence of *Ostrea edulis* RNA was evaluated by real-time (RT)-PCR targeting the oyster EF1α gene as described in Morga et al. (2010). One of the duplicates yielded late amplification (Ct 35.70), suggesting the presence of a low amount of *O. edulis* EF1α transcripts. Finally, the presence of *B. ostreae* RNA was checked by qRT-PCR using primers to amplify the actin gene from Robert et al. (2009), following the same protocol as for EF1α. The detection of the parasite in both duplicates with 19.99 Ct values confirmed the presence of a large amount of *B. ostreae* RNA [which could be estimated at around 1.95 × 10^6^ copies of actin genes if we extrapolate the results of the study by Robert et al. (2009)].

### Illumina Libraries Preparation and Sequencing

Libraries were prepared using the Illumina Tru-Seq RNA-Seq protocol and sequencing was done using a HiSeq2000 Illumina in paired-end 2 × 100 bp and using TruSeq PE Cluster v3 and TruSeq SBS 200 cycle v3 (illumina^®^) kits.

### *De Novo* Transcriptome Assembly and Evaluation

#### Quality Control Verification and Read Classification

**Figure 2:**
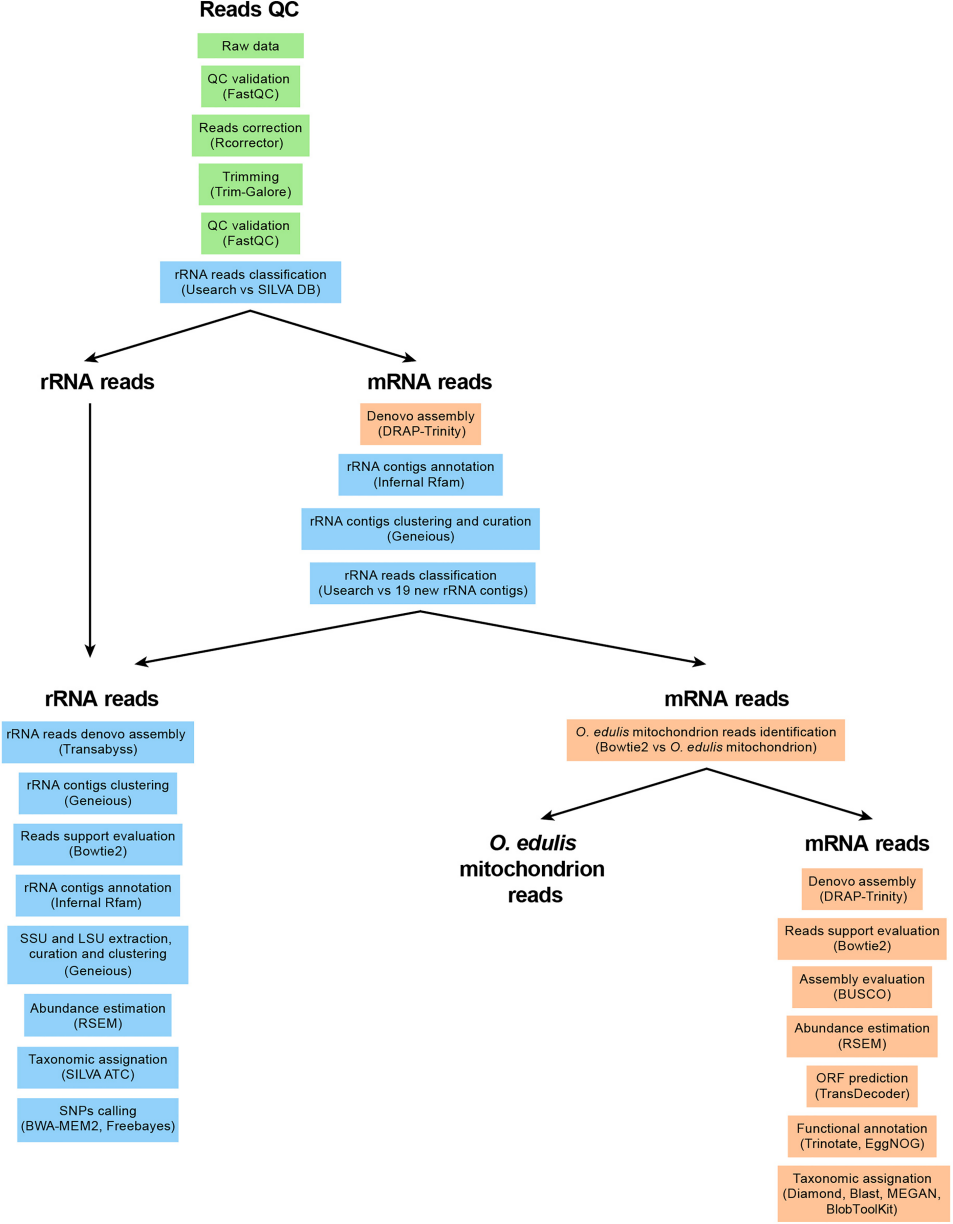
Flowchart of the bioinformatic pipeline. First, QC reads were performed, followed by classification reads as “ribosomal” rRNA reads or “coding” mRNA reads. Finally, each read collections has been assembled and annotated separately with dedicated tools.

Raw sequencing reads were quality checked (**Figure 2**) with FastQC version 0.11.5 (https://github.com/s-andrews/FastQC). The reads were then processed with Rcorrector version 1.0.4 with standard parameters to correct random sequencing errors in Illumina RNA-seq reads. Rcorrector uses a k-mer-based method and a De Bruijn graph to compactly represent all trusted k-mers in the input reads and correct them (Song and Florea, 2015). Corrected reads were then trimmed with Trim-Galore version 0.6.4 with the following parameters: –paired –phred33 –length 36 -q 5 –stringency 1 -e 0.1 –cores 4 (https://github.com/FelixKrueger/TrimGalore). After the last QC verification with FastQC, the quality-filtered (hereafter, good-quality) RNA-Seq reads were organized into two subsets: messenger RNA (mRNA) sequences and ribosomal RNA (rRNA) sequences. To this end, all good-quality RNA reads were searched against the SILVA rRNA database version 138.1 (Quast et al., 2013) with Usearch version 11.0.667 with parameters: -usearch_local -db -id 0.8 -strand both -top_hit_only (Edgar, 2010). All mRNA reads (i.e., reads without a Usearch hit against the SILVA database) were *de novo* assembled with a first run of the *De novo* RNA-Seq Assembly Pipeline (DRAP)-Trinity version 1.92 with standard parameters (Cabau et al., 2017). The new *de novo*-assembled contigs were then searched for potential remaining rRNA transcripts using Infernal version 1.1.3 and the following parameters: -Z –cut_ga –rfam –nohmmonl –fmt 2 (Nawrocki and Eddy, 2013) by identifying covariance model (CM) against the Rfam database (Kalvari et al., 2021). After clustering and manual curation in Geneious Prime version 2020.2.3 (https://www.geneious.com), 19 new rRNA contigs were identified and used as new rRNA custom database for a final read classification with Usearch.

#### rRNA Reads Assembly and Analysis

The rRNA reads identified were *de novo* assembled with Transabyss version 2.0.1 with the following parameters: transabyss -k 25/35/45/55/65; transabyss-merge –mink 25 –maxk 65 –SS (Robertson et al., 2010). The new *de novo* rRNA contigs were then clustered and size filtered (only contig >200 bp were kept for subsequent analysis) in Geneious. High-quality nonredundant rRNA contigs were annotated with Infernal by identifying rRNA CM against the Rfam database. Following rRNA prediction, small subunit (SSU) and large subunit (LSU) rRNA were extracted, curated, and clustered in Geneious. Abundance estimations were then assessed with RSEM *via* the Trinity script align_and_estimate_abundance.pl with the following parameters: –est_method RSEM version –aln_method bowtie2 (https://github.com/trinityrnaseq/trinityrnaseq/blob/master/util/align_and_estimate_abundance.pl). The top ten most expressed SSU and LSU rRNAs were then subjected to BLASTn (Altschul et al., 1990) search against the nt database (accessed in February 2021) followed by a taxonomic assignment with the web-based tool SILVA ATC (https://www.arb-silva.de/aligner) (accessed February 2021) (Pruesse et al., 2012).

#### Identification and Characterization of *B. ostreae* rRNA Operon

The longest and most abundant *de novo*-assembled rRNA transcripts by the Transabyss assembler were subjected to a BLASTn search against nt. Top BLASTn hit allowed us to identify the position of the previously sequenced *B. ostreae* rRNA operon fragment on our new putative full-length *B. ostreae* rRNA operon transcript. In order to characterize the diversity of the *B. ostreae* population in our metatranscriptomic sample, we then performed a variant calling by realigning the rRNA reads to the *B. ostreae* rRNA operon contig with BWA-MEM2 version 2.1 with standard parameter (Li and Durbin, 2010), followed by a variant calling with Freebayes version 1.3.2 with the following parameters: -F 0.01 -C 1 –pooled-continuous –report-monomorphic (https://github.com/freebayes/freebayes). The sequence of this *B. ostreae* rRNA operon has been deposited at NCBI under accession number MZ305451.

#### mRNA Read Assembly and Analysis

First, host (*Ostrea edulis*) mitochondrion reads were identified by mapping mRNA reads against the *O. edulis* mitochondrion genome (accession: JF274008.1) with Bowtie2 version 2.4.1 with the following parameters: –very-sensitive-local (Langmead and Salzberg, 2012). Second, the remaining mRNA reads were *de novo* assembled with a second run of DRAP-Trinity with the same parameters as previously described. Read-support evaluation of the assembled transcriptome was accessed by realigning mRNA reads against the new *de novo*-assembled transcripts with Bowtie2 version 2.4.1 with the following parameter: -k 20. Transcriptome completeness was assessed by submitting transcripts to BUSCO evaluation version 5.0.0 with the following parameters: -m transcriptome on one of the following odb10 databases: Alveolata, Archaea, Bacteria, Eukaryota, Fungi, Metazoa, Mollusca, Stramenopiles, and Viridiplantae (Seppey et al., 2019). Transcript abundance was then estimated with RSEM *via* the Trinity script align_and_estimate_abundance.pl. Transcriptome assembly annotation was performed with Trinotate version 3.1.1 (https://github.com/Trinotate/Trinotate.github.io/wiki). First, DRAP contigs were scanned with Transdecoder version 5.5.0 with standard parameters (https://github.com/TransDecoder/TransDecoder/wiki) for ORF prediction. Functional annotation was then performed by a series of public database interrogations with (i) BLASTx (transcripts) (parameters: -max_target_seqs 1 -evalue 1e-3) and BLASTp (CDS peptide) (parameters: -max_target_seqs 1 -evalue 1e-3) against the Swiss-Prot database (accessed in February 2021), with (ii) hmmscan version 3.3 with standard parameters (Mistry et al., 2013) against the Pfam-A database (accessed in February 2021), with (iii) TmHMM version 2.0c with standard parameters (Krogh et al., 2001), with (iv) SignalP version 5.0b with standard parameters (Nielsen et al., 2019), with (v) eggNOG-mapper version 2 with standard parameters (Huerta-Cepas et al., 2017) against the eggnog v5.0 database (accessed in February 2021) (Huerta-Cepas et al., 2019), and with (vi) InterProScan implemented in ORSON (prOteome and tRanScriptome functiOnal aNnotation) (https://gitlab.ifremer.fr/bioinfo/orson).

#### Transcriptome Taxonomic Assigment

Taxonomic assigment of the whole collection of the *de novo*-assembled transcripts was performed with two different tools: (i) with MEGAN version 6.20.18 (Huson et al., 2016) after a DIAMOND-blastx (version 2.0.6) interrogation of the mRNA transcripts with parameters: –sensitive –max-target-seqs 100 –e value 1e-3 -F 15 –range-culling against the RefSeq protein database, accessed in February 2021 (Buchfink et al., 2021); and (ii) with Blobtool2 implemented in BlobToolKit version 1.3.6 (Challis et al., 2020) after a BLASTn (version 2.9.0) interrogation of the mRNA transcripts against the nt database accessed in February 2021 with the following parameters: -max_hsps 1 -max_target_seqs 10 -evalue 1e-25 and a DIAMOND-blastx interrogation of the mRNA transcripts against the RefSeq protein database with parameters: –sensitive –max-target-seqs 1 –evalue 1e-25 (version 2.0.6, accessed in February 2021).

### ONT Amplicon Sequencing

#### DNA Extraction and PCR Amplification

Gills were dissected from a *B. ostreae* infected flat oyster collected in February 2008 from Cancale Bay (Brittany, France). DNA was extracted using the QIAmp DNA Mini Kit (QIAGEN) and stored in buffer AE at −20°C. This DNA was then used as a PCR template for amplification in March 2021 with primers designed to amplify a large part corresponding to 92% of the *B. ostreae* rRNA operon assembled and identified in the present study (see Identification and Characterization of *B. ostreae* rRNA Operon). The forward sequence primer is 304F-TCGGCGGGAGTGCATATTAG, and the reverse sequence primer is 5457R-ATCTGGGAGAGGGGCTGAAT. Four PCR reactions were performed with 9 µl of ddH_2_O, 12.5 µl of Q5 (NEB) master mix, 1.25 µl of each primer (0.5 µM), and 1 µl of DNA (50 ng/µl). The PCR cycle was set up as follows: starting denaturation at 95°C for 5 min, 30 cycles of denaturation at 95°C for 30 s, annealing at 62°C for 2 min, elongation at 72°C for 5 min, terminated by a final elongation step at 72°C for 5min, followed by storage at 4°C until PCR product purification. The four PCR reactions were subjected to ethanol precipitation. We then used the Qubit dsDNA HS Assay (Thermo Fisher Scientific) to determine the DNA concentrations of the purified PCR products.

#### Library Preparation and ONT Sequencing

In total, 860 ng of purified PCR amplicons were used for sequencing library preparation with the Nanopore genomic sequencing kit SQK-LSK109 (Oxford Nanopore) following the amplicon by ligation protocol (version ACDE_9064_v109_revQ_14Aug2019). Subsequently, 20.76 ng of the prepared library was loaded onto a new flowcell R9 (FLO-MIN106D) with 1,356 pores available. Sequencing was performed on MinION Mk1c for 5 h using the corresponding workflow on MinKNOW.

#### ONT Bioinformatics Analyses

Raw fast5 Nanopore reads were base-called with Guppy (version v4.5.3), then base-called fastq reads were searched for residual Nanopore adapter and PCR primer with PoreChop (version 0.2.4) (https://github.com/rrwick/Porechop) with the following parameters: –adapter_threshold 80 –middle_threshold 80 –extra_middle_trim_bad_side 10. Clean fastq reads were then aligned with minimap2 (version: 2.18) to the full-length *B. ostreae* rRNA operon (accession: MZ305451) assembled and identified in the present study. Finally, the alignment file was finally processed with SAMtools to produce a sorted and indexed bam file, which was subjected to variant calling with the tools PEPPER (https://github.com/kishwarshafin/pepper) (version 0.4) with parameters –ont –gvcf.

## Results

### Sequencing Statistics

The first transcriptome of *Bonamia ostreae* was sequenced from parasites isolated from 2 highly infected flat oysters. This RNAseq generated 183.1M high-quality reads that were classified as 4.2 M rRNA reads, 2.5 M *O. edulis* mitochondrion reads, and 176.3 M mRNA reads (**Table 1**).

**Table 1:** Global statistics of *B. ostreae* transcriptome.

Reads statistics		
Raw reads (#)	188,880,795	
Quality cleaned reads (#)	183,105,338	
rRNA reads (#)	4,209,956	
*O. edulis* mitochondrion reads (#)	2,508,617	
mRNA reads (#)	176,386,765	
**Assembly statistics rRNA reads**		
*De novo* Trans-ABySS contigs (#)	3,189	
Clean rRNA contigs	468	
rRNA subunits (#)	SSU	LSU
	120	219
Average contig (bp)	534.8	606.7
GC %	51.8	52.4
Minimum transcript length (bp)	120	64
Maximum transcript length (bp)	1,853	3,818
Overall Bowtie2 mapping (%)	96.72	
**Assembly statistics mRNA reads**		
DRAP-Trinity contigs (#)	15,618	
Average contig (bp)	1,425.5	
GC%	37.1	
Minimum transcript length (bp)	201	
Maximum transcript length (bp)	11,604	
Overall Bowtie2 mapping (%)	96.97	
BlastX hit (#)	17,945	
**Annotation statistics**		
Transcripts with CDS (#)	13,600	
Predicted CDS (#)	24,995	
Average CDS (bp)	659.3	
Minimum CDS length (bp)	120	
Maximum CDS length (bp)	7917	
GC %	39.2	
EggNOG hit (#)	10,828	
BlastP hit (#)	11,745	
Pfam hit (#)	105,663	

The enrichment process of the parasite from the host tissues might also retain a small number of host cellular debris and other microorganism cells. Therefore, in addition to the controls performed on the RNA suspension, to have a complete overview of the level of nontarget contamination, the analysis of this meta-transcriptome was carried out in two parts: (i) first, we used Transabyss to perform a specific *de novo* assembly of the rRNA reads (see *Materials and Methods*) and (ii) second, we then assembled the mRNA reads with DRAP-Trinity (see *Materials and Methods*).

### Assembly of rRNA Reads

**Figure 3:**
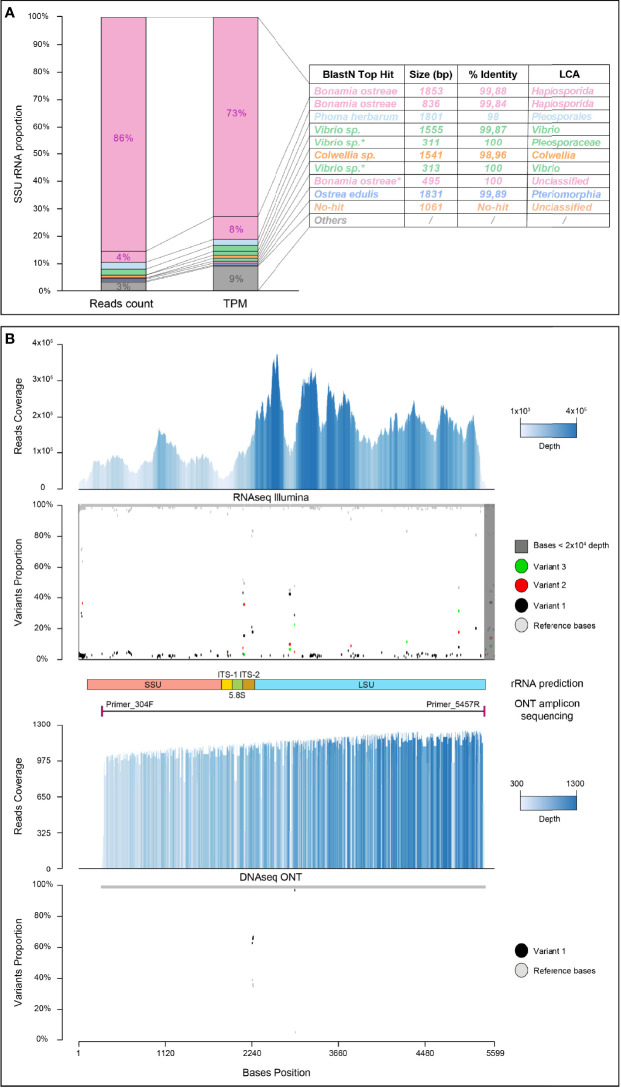
Description of the rRNA transcript population. **(A)** Taxonomic classification of the top ten expressed rRNA transcripts identified using SILVA ATC and BlastN with their respective read counts and expression values. LCA, last common ancestor; TPM, transcripts per million. *Multiple top hits with the same percentage of identity and e-value. **(B)** Characterization of the putative *B. ostreae* rRNA operon. The first panel represents Illumina read coverage on the putative rRNA operon color coded from light blue 1 × 10^3^ read depth to dark blue 4 × 10^5^ read depth. The second panel shows the diversity analysis performed by Freebayes from the Illumina read alignment on the putative operon. The top three variants are color coded in red, blue, and green relative to the reference base in grey. Regions with low read coverage (<2 × 10^4^) were masked (dark grey) and did not have variants called. The third panel highlights the structures of ribosomal subunits predicted by Infernal and the positions of PCR primers used for nanopore amplicon sequencing. The fourth panel represents ONT read coverage on the putative rRNA operon, color coded from light blue 300 read depth to dark blue 1,300 read depth. The fifth panel shows the diversity analysis performed by PEPPER from the ONT read alignment on the putative operon. Variants are color coded in red relative to the reference base in grey.

We decided to run the *de novo* assembly of the rRNA reads with the Transabyss assembler because it has been shown to be more accurate at assembling non-coding RNA reads (Lau et al., 2018). We eventually assembled 3189 rRNA contigs that we have clustered and manually curated in Geneious to finally obtain 468 nonredundant rRNA transcripts (**Table 1**). We considered those 468 rRNA transcripts as highly representative of the rRNA population of our transcriptome since 96.72% of our identified rRNA reads were aligned back to our *de novo*-assembled rRNA transcripts (**Table 1**). To accurately determine the taxonomic classification of these rRNA transcripts, we then predicted and extracted both SSU and LSU from all rRNA transcripts and submitted each collection of SSU or LSU to SILVA ATC for taxonomic classification. Since the SILVA SSU database is more complete and accurate than the LSU database, we have focused our SILVA taxonomic classification results on the SSU transcripts. This SILVA classification, along with BlastN top hit identification, showed that more than 90% of the SSU rRNA reads aligned to SSU rRNA transcripts belonging to the order *Haplosporida* and more precisely to *B. ostreae* (**Figure 3A**).

We then wanted to obtain a complete overview of the *B. ostreae* diversity in our parasite-enriched sample. To this end, we first identified the putative full-length *B. ostreae* rRNA operon (see *Materials and Methods*) and used it as a reference for read mapping and variant calling (see *Materials and Methods*). Our *B. ostreae* rRNA operon diversity analysis shows that the majority of the rRNA operon sequences are either identical or show minor diversity in our samples. However, some nucleotide positions show a high level of diversity, suggesting that different populations of *B. ostreae* could cohabit inside the same host batch (**Figure 3B**). Nevertheless, as the *B. ostreae* reference genome is not yet available, we cannot exclude the possibility that the observed diversity is due to the presence of multicopy operons within *B. ostreae* genome (**Figure 3B**). Moreover, as the complete lifecycle of *B. ostreae* is not yet fully characterized, the ploidy state of the parasite is not known; therefore, this diversity could also be due to the presence of heterozygous alleles in the case of a polyploid genome.

Finally, to confirm the sequence and the structure of the newly identified *B. ostreae* full-length rRNA operon, we have designed a PCR primer targeting 92% of the rRNA operon (5,173 bp), amplified this fragment from another infected flat oyster individual sampled in 2008, and sequenced it with nanopore technology. A total of 10,100 bases called nanopore reads, corresponding to 15.4 million bases, were sequenced. Among them, 1,573 reads with an average size of 4,048 bp, a GC% of 52.4%, and composed of 6 million sequenced bases were aligned to the *B. ostreae* operon, leading to a mean coverage of 880 reads. In contrast, the 8,067 unaligned reads showed an average size of 1,089 bp, a GC% of 35%, and a total of 9.4 million sequenced bases. Those unaligned reads were mostly assigned to the non-rRNA region of *O. edulis* (using the draft genome of *O. edulis* data not shown). This result can be explained by the fact that a total of 200 ng of old *O. edulis* DNA (12 years stored at −20°C) was used for the four LR-PCR reactions used to amplify the *B. ostreae* rRNA operon. As nanopore sequencing is known to promote the sequencing of small DNA molecules, this could have resulted in a large number of small *O. edulis* DNA molecules being sequenced during the nanopore sequencing run. Variant calling performed with PEPPER identified only one SNP (A to G) in position 2334 (ITS-2) with a frequency of 62%, one deletion of eight nucleotides (CCTCGTAA) in position 2339 (ITS-2) with a frequency of 66%, and one SNP (T to A) in position 2914 (LSU) with a frequency of 98% (**Figure 3B**).

Since this former variation is probably fixed between the *B. ostreae* sampled in 2012 (RNAseq) and the ones sampled in 2008 (DNA amplicon sequencing), the two observed variations in the ITS-2 may be related to modifications of putative different copies of the rRNA operon present in the *B. ostreae* genome or the presence of heterozygous alleles.

### Assembly of mRNA Reads

**Figure 4:**
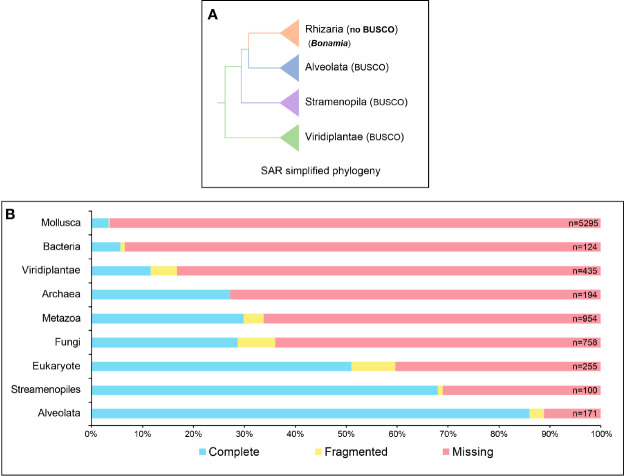
BUSCO transcriptome evaluation. **(A)** Simplified Stramenopila, Alveolata, and Rhizaria (SAR) phylogeny adapted from Sierra et al. (2016). The Rhizaria clade does not have a proper BUSCO database due to the lack of genomic data in this clade, whereas the closely related clades Alveolata and Stramenopila are represented by proper BUSCO databases. **(B)** BUSCO evaluation of the *B. ostreae* transcriptome. This evaluation has been performed by interrogating a large panel of different database clades related or not to *B. ostreae* and its host, *O. edulis*.

**Figure 5:**
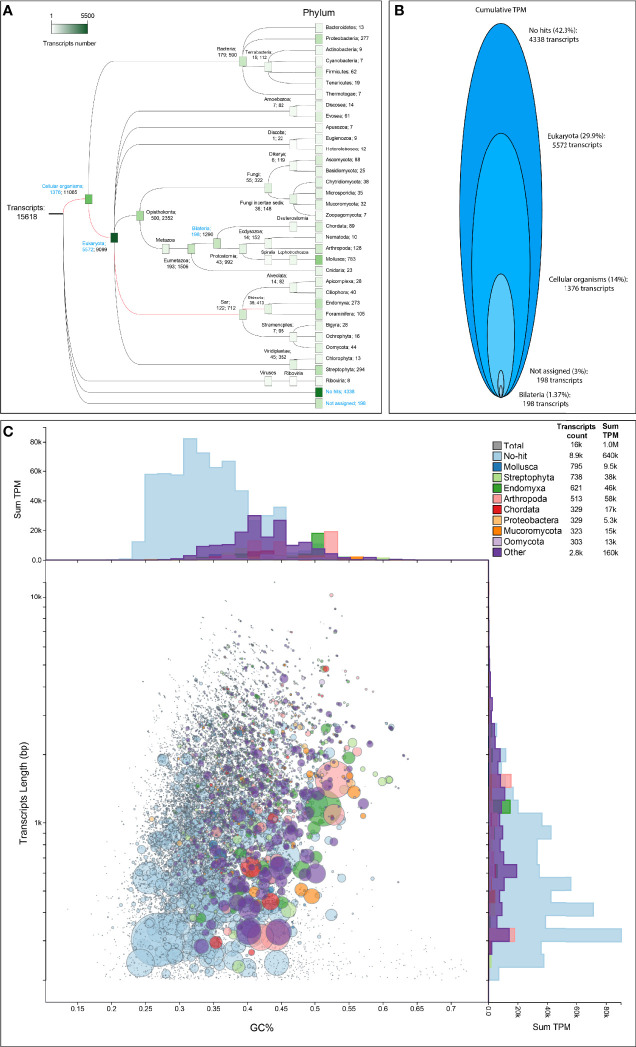
Whole transcriptome taxonomic assignment. **(A)** Taxonomic assignment of transcripts using MEGAN following a DIAMOND-BlastX interrogation of the RefSeq database. The first number for each clade corresponds to the number of transcripts assigned to this clade, whereas the second number corresponds to the number of transcripts assigned to the subsequent clades. Each clade is color coded from white (at least one transcript is assigned) to dark green (5,500 transcripts are assigned). Red branches correspond to the phylogenetic path to the Rhizaria clade. Blue clades correspond to clades for which cumulative expression has been plotted in **(B)**. **(C)** Taxonomic assignment of transcripts performed with BlobToolKit after a DIAMOND-BlastX interrogation of the RefSeq database and a BlastN interrogation of the nt database. A blob plot of length versus GC proportion for each transcript was created. Records are colored by phylum. Circles are sized in proportion to TPM on a square-root scale. Histograms show the distribution of TPM sum along each axis.

Assembly of the mRNA reads was done with the assembly pipeline DRAP-Trinity (see *Materials and Methods*). This pipeline allows us to increase the compactness of the assembly, reduce the chimerism of the *de novon*- assembled contigs, and improve the error rates of the whole transcriptome (Cabau et al., 2017). Finally, we were able to *de novo* assemble 15,618 high-quality transcripts with an average size of 1,425.5 bp and a GC% of 37.1% (**Table 1**). As observed for the rRNA assembly, we are confident that our mRNA assembly is highly representative of our transcriptome since 96.97% of our identified mRNA reads were aligned back to our *de novo*-assembled mRNA transcripts (**Table 1**). Furthermore, BUSCO analysis of our *de novo*-assembled transcriptome identified the group Alveolata as the most complete BUSCO. Since the group Rhizaria to which *B. ostreae* belongs does not have its own BUSCO database and that Alveolata is the closest group of the Rhizaria inside the SAR clade, we believe that our transcriptome is mainly represented by transcripts closely related to protozoa belonging to SAR clade and very likely to *B. ostreae* (**Figure 4**). To obtain a global overview of the taxonomic range of our transcriptomic dataset, we then performed a taxonomic classification of the 15,618 identified transcripts with two different tools: MEGAN and BlobToolsKit (see *Materials and Methods*). The MEGAN taxonomic classification identified that more than 90% of the whole cumulative expression of our transcriptome measured as transcripts per million (TPM) is represented by 11,682 transcripts distributed as follows: 4,338 transcripts with no hits in the RefSeq protein database, 5,572 transcripts belonging to Eukaryota, 1,376 transcripts belonging to a cellular organism, 198 transcripts that could not be assigned to any taxonomic group by MEGAN (usually when a transcript have several Blast hits with the same evalue) and 198 transcripts assigned to Bilateria (**Figures 5A, B** **)**. The BlobToolKit taxonomic classification showed a similar trend with most of the cumulative expression (>60%) represented by transcripts with no hit in the interrogated databases (**Figure 5C**). Interestingly, this blobplot showed that the transcripts with no hit tend to have a lower GC% than the transcripts harboring a hit with a taxonomy assignment (**Figure 5C**). These results support the hypothesis that our *B. ostreae* transcriptome is mainly represented by transcripts without homology to other sequences present in the interrogated database, and this emphasizes the need to obtain more reference sequences for *B. ostreae* and other protozoa from the Rhizarian clade.

### Annotation of mRNA Transcripts

**Figure 6:**
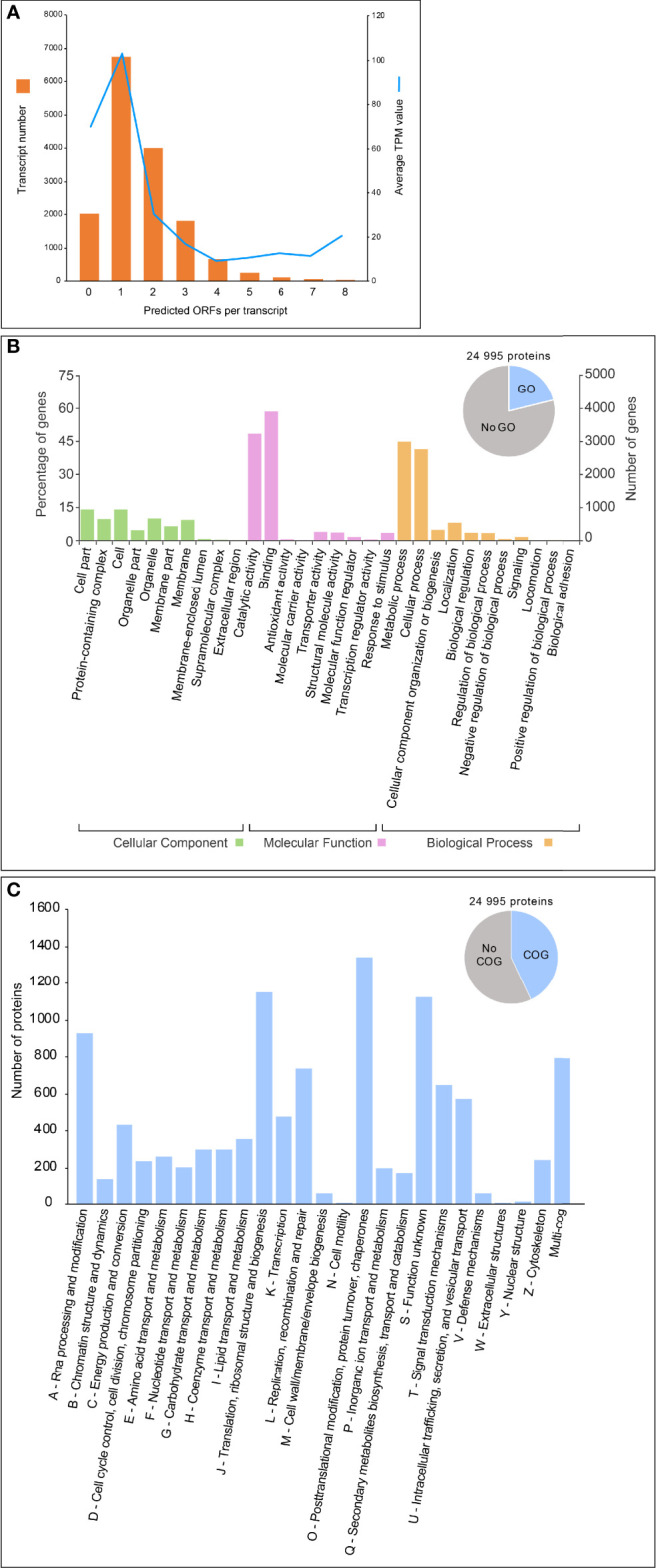
Whole transcriptome functional annotation. **(A)** Number of predicted ORFs for each transcript. Orange histograms represent the number of transcripts for each number of predicted ORFs. Blue line denotes the average TPM value for each number of predicted ORFs. **(B)** GO functional annotation as determined by InterProScan annotation. **(C)** COG functional annotation assessed by EggNOG-mapper.

ORF prediction performed by Trinotate on the 15,618 transcripts identified 24,995 putative peptides. The majority of transcripts possess one ORF (6,740 transcripts) or two ORFs (3,992 transcripts), and a maximum of 8 ORFs per transcript (6 transcripts) have been observed (**Figure 6A**). Even if the DRAP pipeline utilized here for the *de novo* assembly tends to reduce the creation of spurious contigs (i.e., chimeric contig), some transcripts could have been misassembled during the pipeline. As our multiple taxonomic assignments showed, several of those polycistronic transcripts could result from the transcription of prokaryotic operons expressed by some of the bacterial contamination observed in our sample. However, as the reference genome of *B. ostreae* is not yet available, we could not reject the hypothesis that many *B. ostreae* genes are true polycistronic transcripts. Additionally, 2,018 transcripts do not contain any ORFs (**Figure 6A**), these orphan transcripts likely represent other populations of noncoding RNA different from the rRNAs (i.e., lncRNA, snRNA, snoRNA) or correspond to genes undergoing pseudogenization for which transcript is still transcribed but not translated into functional protein (Wen et al., 2012). Finally, it is important to mention that transcripts with one ORF showed the highest average TPM expression value (>100), meaning that transcripts with one ORF are probably less spurious than other transcripts (**Figure 6A**).

The 24,995 identified proteins have then been extensively annotated through several database interrogations (see *Materials and Methods*). Among them, 6,673 protein-coding transcripts were mapped to GO terms from InterProScan similarity hits, with the most represented terms belonging to “catalytic activity,” “binding,” “metabolic process,” and “cellular process” (**Figure 6B**). Furthermore, 9,950 proteins were assigned to putative functional categories using the Clusters of Orthologous Groups of proteins (COG) database from the EggNOG mapper (**Figure 6C**). The most representative COG function includes “A-RNA processing,” “J-Translation,” and “O-Post-translational modification,” while the less representative COG function includes “W-Extracellular structure,” “Y-Nuclear structure,” and “N-Cell motility” (**Figure 6C**).

This functional annotation gives some insight into the metabolic activity of *B. ostreae* and confirms the active function related to its intracellular lifestyle.

The observed functional annotation was further validated by a fine analysis of the top 30 expressed transcripts, which represent more than 20% of the cumulative expression measured as TPM (**Table 2**). Indeed, among those 30 transcripts, 6 transcripts do not harbor any ORF and do not have similarities with the Rfam database, 6 transcripts possess a predicted ORF but without identified functional annotation, and 12 transcripts code for a protein with functions involved in transcription and translation, which reflects an important biological activity (**Table 2**).

**Table 2:** Description of the top 30 expressed transcripts.

Transcript ID	Predicted function (Trinotate/eggNOG)	COG classification (eggNOG)	Predicted ORF (TransDecoder)	Length	Effective length (RSEM)	Expected count (RSEM)	Cumulative count (RSEM)	Cumulative count % (RSEM)	TPM (RSEM)	Cumulative TPM (RSEM)	Cumulative TPM % (RSEM)	LCA (Diamond-Blastx/MEGAN)
BO_RNAseq_Transcript_009714	No hit/no predicted ORF	–	0	302	133.39	940,211.00	940,211.00	0.58%	29,256.51	29,256.51	2.93%	No hits
BO_RNAseq_Transcript_007095	No hit/no predicted ORF	–	0	298	129.39	769,231.00	1,709,442.00	1.05%	24,675.95	53,932.46	5.39%	No hits
BO_RNAseq_Transcript_004247	No hit/no predicted ORF	–	0	440	271.38	1,093,205.00	2,802,647.00	1.72%	16,720.50	70,652.96	7.07%	No hits
BO_RNAseq_Transcript_013065	EF1a	J	1	1,549	1,380.37	4,797,256.00	7,599,903.00	4.67%	14,425.06	14,425.06	1.44%	Cellular organisms
BO_RNAseq_Transcript_010383	Actin-3	Z	1	1,159	990.37	3,206,407.11	10,806,310.11	6.64%	13,438.20	27,863.26	2.79%	Cellular organisms
BO_RNAseq_Transcript_001385	No hit	–	1	934	765.37	2,237,662.77	13,043,972.88	8.02%	12,135.08	39,998.34	4.00%	No hits
BO_RNAseq_Transcript_001065	Zinc finger	–	1	704	535.37	1,554,114.00	14,598,086.88	8.97%	12,048.89	52,047.23	5.20%	No hits
BO_RNAseq_Transcript_010875	RPS21e	J	1	331	162.39	416,025.00	15,014,111.88	9.23%	10,633.90	62,681.13	6.27%	Cellular organisms
BO_RNAseq_Transcript_005076	No hit	–	1	569	400.37	997,611.00	16,011,722.88	9.84%	10,342.27	73,023.40	7.30%	No hits
BO_RNAseq_Transcript_000203	PBZ and calponin domain	–	2	1,096	927.37	2,213,827.53	18,225,550.41	11.20%	9,908.56	82,931.96	8.29%	No hits
BO_RNAseq_Transcript_000134	Ubiquitin	O	1	315	146.39	321,668.24	18,547,218.65	11.40%	9,120.61	92,052.57	9.21%	Cellular organisms
BO_RNAseq_Transcript_010816	No hit	–	1	239	70.41	134,922.00	18,682,140.65	11.48%	7,953.37	100,005.94	10.00%	No hits
BO_RNAseq_Transcript_005904	No hit/no predicted ORF	–	0	284	115.39	209,666.00	18,891,806.65	11.61%	7,541.62	107,547.56	10.75%	No hits
BO_RNAseq_Transcript_006793	Ubiquitin	O	1	310	141.39	245,151.76	19,136,958.41	11.76%	7,196.83	114,744.39	11.47%	Cellular organisms
BO_RNAseq_Transcript_008363	RPL37e	J	1	327	158.39	270,502.00	19,407,460.41	11.93%	7,088.82	121,833.21	12.18%	Eukaryota
BO_RNAseq_Transcript_010422	No hit	–	1	446	277.38	469,934.00	19,877,394.41	12.21%	7,032.14	128,865.35	12.89%	No hits
BO_RNAseq_Transcript_001129	Poly(A)-binding protein 4	A	1	1,209	1,040.37	1,715,543.13	21,592,937.54	13.27%	6,844.37	135,709.72	13.57%	Bilateria
BO_RNAseq_Transcript_001979	No hit/no predicted ORF	–	0	472	303.38	493,676.00	22,086,613.54	13.57%	6,754.33	142,464.05	14.25%	No hits
BO_RNAseq_Transcript_003453	Plant cadmium resistance	–	0	614	445.37	706,783.42	22,793,396.96	14.01%	6,586.92	149,050.97	14.91%	Eukaryota
BO_RNAseq_Transcript_006534	CD34/podocalyxin	–	1	515	346.38	545,905.00	23,339,301.96	14.34%	6,541.71	155,592.68	15.56%	No hits
BO_RNAseq_Transcript_011023	MA3 domain-containing protein 6	–	1	239	70.41	109,433.00	23,448,734.96	14.41%	6,450.85	162,043.53	16.20%	Eukaryota
BO_RNAseq_Transcript_007490	RPL43	J	1	326	157.39	239,573.00	23,688,307.96	14.56%	6,318.18	168,361.71	16.84%	Eukaryota
BO_RNAseq_Transcript_004224	EPSP_synthase	–	1	400	231.38	312,025.00	24,000,332.96	14.75%	5,597.38	173,959.09	17.40%	Dothideomycetes
BO_RNAseq_Transcript_002381	RPS7e	J	1	629	460.37	617,846.00	24,618,178.96	15.13%	5,570.46	179,529.55	17.95%	Eukaryota
BO_RNAseq_Transcript_010230	No hit/no predicted ORF	–	0	356	187.38	236,895.00	24,855,073.96	15.27%	5,247.43	184,776.98	18.48%	No hits
BO_RNAseq_Transcript_004899	RPL35a	J	1	415	246.38	295,389.00	25,150,462.96	15.45%	4,976.36	189,753.34	18.98%	Eukaryota
BO_RNAseq_Transcript_000954	No hit	–	1	465	296.38	346,556.23	25,497,019.19	15.67%	4,853.46	194,606.80	19.46%	No hits
BO_RNAseq_Transcript_010468	No hit	–	1	1,029	860.37	987,367.47	26,484,386.66	16.27%	4,763.36	199,370.16	19.94%	No hits
BO_RNAseq_Transcript_006985	RPS14	J	1	637	468.37	512,314.27	26,996,700.93	16.59%	4,540.10	203,910.26	20.39%	NCBI
BO_RNAseq_Transcript_012092	RPS5	J	2	1,083	914.37	994,736.52	27,991,437.45	17.20%	4,515.50	208,425.76	20.84%	Eukaryota

## Discussion

This is the first transcriptome of the pathogenic protozoa *B. ostreae* involved in the important decline of the European native flat oyster populations since the 1980s (Pogoda et al., 2019). Because this protozoan is not yet culturable, it remains extremely challenging to obtain high-quality *-omic* data, and to date, no genomic nor transcriptomic data are available except for small rRNA or actin gene fragments (see Background and Summary).

Currently, the only way to obtain sufficient biological material for this protozoa is to perform an enrichment/isolation of the parasitic cells from highly infected *O. edulis* hosts. Unfortunately, this process does not lead to 100% *B. ostreae*-containing samples.

The present *B. ostreae* transcriptome demonstrates that the enrichment approach is efficient since most transcripts belong to *B. ostreae*. However, the presence of transcripts from other organisms reflects the difficulty of obtaining a pure *B. ostreae* sample. Thus, we decided to process this transcriptome as a meta-transcriptome and divide the analysis into two main parts: (i) the evaluation of the rRNA population and (ii) the evaluation of mRNA.

Indeed, even if the molecular biology procedure used here was not designed to sequence rRNA molecules since the cDNAs were produced by polyT-based library preparation, we were able to identify 2.3% of rRNA reads in our dataset. The evaluation of this rRNA population allowed us to confirm that the great majority of rRNAs present in our dataset corresponds to *B. ostreae* [>80% of the total rRNA expression (TPM)].

Furthermore, the extensive functional analysis performed on the putative protein-coding-containing transcripts assembled from mRNA reads showed that the majority of this meta-transcriptome codes for proteins with no homology with proteins belonging to the current major protein database. Moreover, most of the cumulative expression observed in this meta-transcriptome corresponds to transcripts with no hits in those databases or to transcripts without precise taxonomic assignment. This observation emphasizes the lack of *-omic* data regarding the Rhizarian clade and, more specifically, regarding *B. ostreae* in a public biological database.

This transcriptome is a highly valuable data source to start characterizing the complex biology of this intracellular parasite. The transcriptome has some limitations associated with difficulties in obtaining pure parasite, but the most complete dataset available is likely to prove extremely useful. Additionally, those data will help to develop a new diagnostic technique to improve the identification of *B. ostreae* in complex mollusc samples.

Thanks to this dataset, we were able to assemble the putative full-length *B. ostreae* rRNA operon and confirm its sequence and structure with nanopore amplicon sequencing. This first attempt at characterization of the full *B. ostreae* rRNA operon by nanopore sequencing on long-stored DNA sample (>12 years at −20°C) is extremely promising but requires more development in order to be used as a potential new tool to detect and characterize *B. ostreae* population.

Finally, the bioinformatic approach developed in the present study will benefit the scientific community who studies host–pathogen interactions involving microeukaryotic intracellular parasites without a reference genome. Indeed, this pipeline allows us to accurately evaluate host and nontarget contamination thanks to a fine analysis of the rRNA read population and to evaluate coding transcripts for taxonomic and functional classification.

## Code Availability

The specific commands used to analyze RNA-seq data and to draw the article figures are available at https://gitlab.ifremer.fr/gc7ad08/bonamia-ostreae-rna-seq-data-analysis.

## Data Availability Statement

The datasets presented in this study can be found in online repositories. The names of the repository/repositories and accession number(s) can be found below: https://www.ncbi.nlm.nih.gov/, PRJNA731671 https://www.ncbi.nlm.nih.gov/genbank/, MZ305451 https://figshare.com/, https://doi.org/10.6084/m9.figshare.16592480.v1 https://figshare.com/, https://doi.org/10.6084/m9.figshare.16598633.v1.

## Author Contributions

IA designed the study and conducted the sampling and the RNAseq library preparation. BC monitored flat oysters and carried out parasite isolations. DS and AD-M conducted the nanopore amplicon sequencing. GC performed the bioinformatics analysis. IA and GC wrote the manuscript. All authors listed have made a substantial, direct, and intellectual contribution to the work and approved it for publication.

## Funding

This work was supported by Interreg SUDOE “Aquagenet” (SOE2/P1/E287) and partly by EU DG SANTE in the frame of the European Union Reference Laboratory for Mollusc Diseases.

## Conflict of Interest

The authors declare that the research was conducted in the absence of any commercial or financial relationships that could be construed as a potential conflict of interest.

## Publisher’s Note

All claims expressed in this article are solely those of the authors and do not necessarily represent those of their affiliated organizations, or those of the publisher, the editors and the reviewers. Any product that may be evaluated in this article, or claim that may be made by its manufacturer, is not guaranteed or endorsed by the publisher.
